# The New Pharmacopœia

**Published:** 1880-09

**Authors:** 


### THE NEW PHARMACOPŒIA.

The Pharmacopœia Committee for the decimal revision for 1880, has grave questions presented, touching changes and additions to be made. The members are at work on the new standard of nomenclature, preparations, etc., for the United States, and the medical press should give expression to popular views on the subjects under consideration.

The revision of 1870 made some innovations which seem reasonable and convenient, but this fact should not encourage the present committee to pursue this line to an unwarrantable extent. Chemists have made a radical change in nomenclature and established it amongst themselves within the last decade, which medical men do not heartily approve. We, who were taught chemistry thirty or forty years ago, know comparatively little of it by the present nomenclature, and will not willingly be forced to express chemical facts in the new terms. In fact it involves more study than we can conveniently bestow at our time of life, to learn the new language of chemistry. As to the adoption of the metric system of weights and measures, which is also proposed for adoption by the committee, there seems to be a pressing necessity. A system universally among all civilized nations, we believe is demanded, since medical literature from each other is distributed to all others constantly. It is true that like the new nomenclature in chemistry, time will be required to familiarize physicians and apothecaries with the system. Then our exchanges reveal propositions made to the committee which if adopted will prove still more onerous. Terms peculiar to the German language are proposed for this standard of pharmacy. While continental Europe affords substantial advancement in medicine, we do not see the practical advantages to be derived from their researches, which some claim for them. The great object of the medical sciences is to ameliorate and cure disease, and beautiful theories which do not lead to these results are useless. There is very little to learn, however, and the simplicity of the system and the ease with which it is practiced, aside from the necessity above referred to, make it generally acceptible to physicians who have given the subject much attention.

